# Regression of taxane-related cystoid macular edema after topical dorzolamide treatment: two case reports

**DOI:** 10.1186/s13256-021-02954-8

**Published:** 2021-07-21

**Authors:** Mitsuru Otsubo, Reiko Kinouchi, Takayuki Kamiya, Akitoshi Yoshida

**Affiliations:** 1 Department of Ophthalmology, Asahikawa Medical University, 2-1-1-1 Midorigaoka Higashi, Asahikawa, Hokkaido Japan; 2 Wakkanai City Hospital, 4-11-6 Chuuou, Wakkanai, Hokkaido Japan; 3 Medicine and Engineering Combined Research Institute, Asahikawa Medical University, 2-1-1-1 Midorigaoka Higashi, Asahikawa, Hokkaido 078-8510 Japan

**Keywords:** Dorzolamide, Nab-paclitaxel, Cystoid macular edema, Optical coherence tomography, Taxane

## Abstract

**Background:**

Cystoid macular edema is a rare, vision-threatening side effect of the taxane family of anticancer agents. There is no established treatment or standard treatment protocol for taxane-related cystoid macular edema. Here, we report two cases of taxane-related cystoid macular edema that were treated with topical dorzolamide.

**Case presentation:**

In case 1, a 72-year-old Japanese woman with bilateral geographic choroiditis reported for a follow-up visit with a complaint of blurred vision in both eyes for 2 months after starting nanoparticle albumin-bound paclitaxel chemotherapy for multiple metastases of her breast cancer. Her best-corrected visual acuity had dropped from 1.2 to 0.9 in the right eye and from 1.0 to 0.4 in the left eye. Fundus examination showed no newly active geographic choroiditis lesion, but optical coherence tomography exhibited cystoid macular edema. We suspected taxane-related cystoid macular edema and terminated nanoparticle albumin-bound paclitaxel, and started topical dorzolamide treatment. Cystoid macular edema nearly resolved within 6 weeks in the right eye and within 10 weeks in the left eye after starting topical dorzolamide treatment. The resolution of cystoid macular edema without leaving a chorioretinal scar after discontinuation of paclitaxel confirmed our initial diagnosis of taxane-related cystoid macular edema. A few inconspicuous cystoid spaces persisted at the parafovea for a year after dorzolamide treatment ended, but regressed after restarting dorzolamide treatment without any side effects. Best-corrected visual acuity improved to 1.2 in the right eye and 1.0 in the left eye. In case 2, a 70-year-old Japanese man, who received nanoparticle albumin-bound paclitaxel for pancreatic cancer with multiple metastases, developed bilateral cystoid macular edema. Best-corrected visual acuity was 0.3 bilaterally. Cystoid macular edema resolved within 5 weeks after stopping nanoparticle albumin-bound paclitaxel and starting topical dorzolamide treatment confirming the diagnosis of taxane-related cystoid macular edema. Nine weeks later, best-corrected visual acuity improved to 0.8 in the right eye and 1.0 in the left eye.

**Conclusions:**

Cystoid macular edema in each case resolved within a few months without any side effects using topical dorzolamide and terminating taxane-based chemotherapy. Topical dorzolamide appears to be a safe and effective treatment option for patients with taxane-related cystoid macular edema whose quality of life is threatened by visual disturbances.

## Background

Paclitaxel, a member of the taxane family of anticancer agents, acts by stabilizing cellular microtubules, thus inhibiting cell division [1–3]. Nanoparticle albumin-bound paclitaxel (nab-PTX) is a solvent-free form of paclitaxel used for the treatment of malignant tumors such as breast cancer, lung cancer, and pancreatic cancer. Patients treated with nab-PTX experience fewer side effects than those treated with solvent-based paclitaxel, such as hypersensitivity reactions, febrile neutropenia, and fatigue [4].

Cystoid macular edema (CME) is a side effect that has been reported in less than 10% of patients treated with nab-PTX, and it usually affects bilateral central vision. While the causes of taxane-related CME are unclear, subtle capillary leakage and effect of taxane toxicity on Müller cells or the retinal pigment epithelium (RPE) have been suspected to play a role [5–12]. Although several treatment options have been reported for taxane-related CME, including oral carbonic anhydrase inhibitors (CAIs) [10], nonsteroidal antiinflammatory drugs [13], sub-Tenon’s injection of triamcinolone acetonide [14], intravitreal bevacizumab [15], and pentoxifylline [9], their effectiveness in resolution of CME remains to be proven [6–9]. Dorzolamide, a topical CAI available as eye drops, is used to lower the intraocular pressure in glaucoma and ocular hypertension; some reports suggest that topical dorzolamide is also effective against taxane-related CME [11, 12, 15]. Shortening the period of visual disturbance without physical burden can improve the quality of life in patients being treated for malignancy with this drug. Here we report two cases of taxane-related CME that resolved after topical dorzolamide treatment. We also present their optical coherence tomography (OCT) images and describe their clinical course.

## Case presentation

Case 1: A 72-year-old Japanese woman with bilateral geographic choroiditis reported to our hospital for a follow-up visit with a complaint of blurred vision after starting nab-PTX. Two months prior, she started receiving chemotherapy with 310 mg nab-PTX (220 mg/m^2^ body surface area) every 3 weeks for breast cancer with bone, pulmonary, and lymph node metastases. She received three treatments with nab-PTX, and visited us 2 weeks after the last dose. She had a history of surgery for thyroid cancer at the age of 53 years and for breast cancer at the age of 58 years. There was no family history of cancer and hereditary ophthalmic disease.

**Fig. 1.**
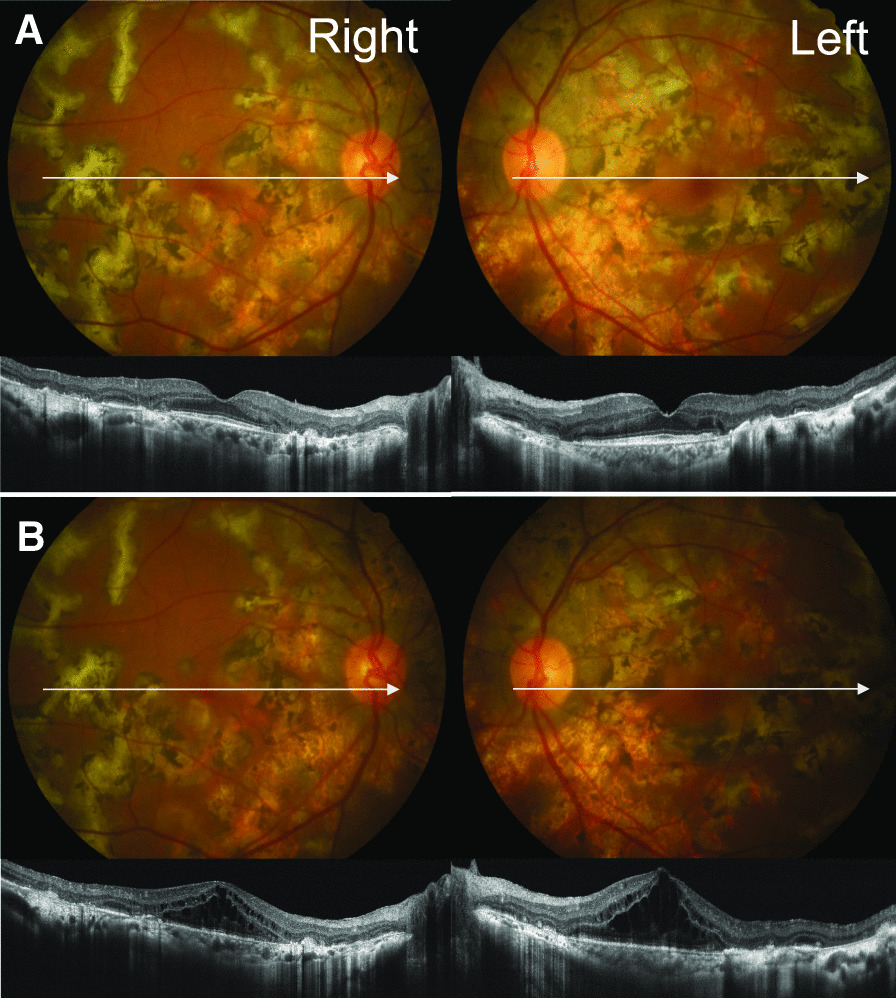
Fundus photographs and optical coherence tomography (OCT) images of case 1 before and after starting nanoparticle albumin-bound paclitaxel (nab-PTX) treatment. **A**, **B** Upper images are fundus photographs. Lower images are 8-mm B scan OCT (RTVue XR Avanti, Optovue, Fremont, CA) images. White arrows in the fundus photographs indicate scanning lines of the OCT shown below those. **A** Images of the right and left eye prior to starting nab-PTX treatment. Fundus photographs show chorioretinal scars from geographic choroiditis. OCT images show scars in the outer retina and choroid. No obvious cystoid macular edema (CME) is observed. **B** Images following three cycles of nab-PTX treatment, 2 weeks after the last treatment. Fundus photographs show no obvious change as compared with pretreatment photographs in (**A**). OCT images show bilateral CME with cystoid spaces located in the inner and outer nuclear layers

Her best-corrected visual acuity (BCVA) declined from 1.2 to 0.9 (decimal visual acuity measured by Landolt C) in the right eye and from 1.0 to 0.4 in the left eye, as compared with the BCVA at the previous visit. The anterior chamber was clear bilaterally, and fundus examination showed no new geographic choroiditis lesions (Fig. 1). OCT showed CME with loss of foveal depression. No obvious CME was seen at the previous visit (Fig. 1).

**Fig. 2.**
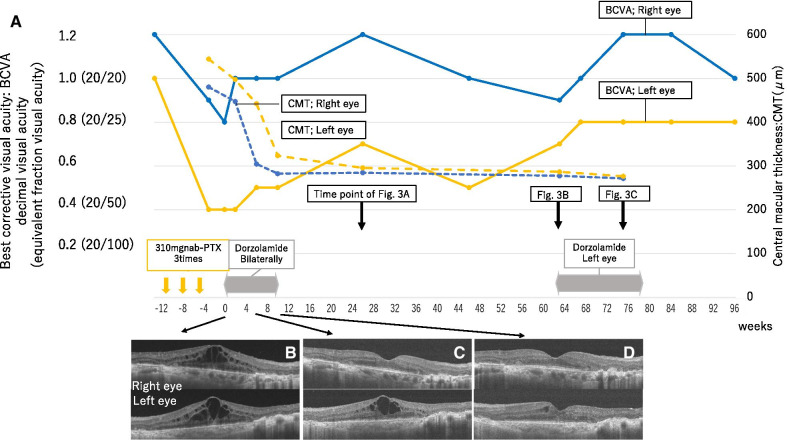
Clinical course of case 1. **A** Changes in best-corrected visual acuity (BCVA) and central macular thickness (CMT) are shown, in addition to the administration period of nanoparticle albumin-bound paclitaxel (nab-PTX) and topical dorzolamide. The numbers on the horizontal line show weeks since topical dorzolamide first started. Time points of each OCT images taken are indicated by arrows. **B**–**D** Optical coherence tomography (OCT) images before and after topical dorzolamide treatment. A 6-mm horizontal sectional OCT scan (**A**: DRI OCT Triton, Topcon, Tokyo, Japan; **B** and **C**: RTVue XR Avanti, Optovue, Fremont, CA). Upper images are of the right eye and lower images are of the left eye. **B** OCT image when dorzolamide treatment was started, 3 weeks after the first visit for blurred vision and 5 weeks after the last nab-PTX treatment. Cystoid macular edema (CME) is observed bilaterally. **C** Six weeks after topical dorzolamide treatment was started. While CME is almost resolved and the foveal depression is restored in the right eye, CME is still observed in the left eye. **D** Ten weeks after topical dorzolamide was started. Foveal depression is restored bilaterally, and few cystoid spaces are observed

Since she was treated with nab-PTX, taxane-related CME was suspected. After consultation with her oncologist, chemotherapy was terminated (Fig. 2A). Because CME persisted for 3 weeks (5 weeks since the last nab-PTX treatment) (Fig. 2B), topical 1% dorzolamide treatment three times daily in both eyes was initiated. CME nearly resolved within 6 weeks in the right eye and within 10 weeks in the left eye after starting topical dorzolamide treatment (Fig. 2C, D); the treatment was then discontinued. Our initial diagnosis of taxane-related CME was confirmed, since CME resolved without chorioretinal scaring and without the administration of immunosuppression therapy.

**Fig. 3.**
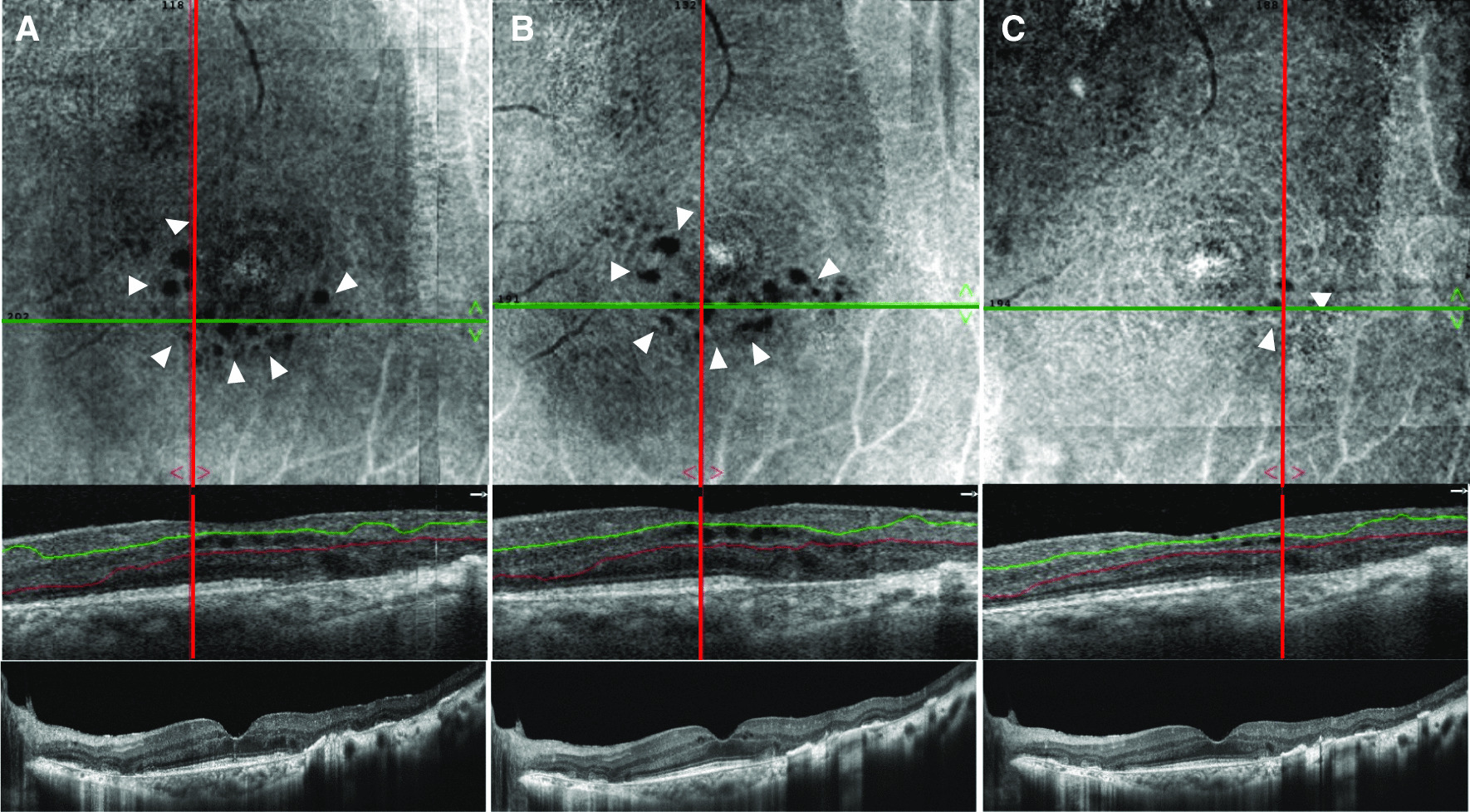
Optical coherence tomography (OCT) images of the left eye in case 1, before and after restarting topical dorzolamide. **A**, **B**, and **C** Upper images are 3 × 3 mm deep layer en face OCT scans. Middle images are 3-mm horizontal sectional scans with segmentation lines for the upper en face OCT images. Lower images are 8-mm horizontal sectional scans across the fovea (RTVue XR Avanti, Optovue, Fremont, CA). White arrows indicate cystoid spaces in the retina. **A** Four months after topical dorzolamide treatment ended. While en face OCT reveals cystoid spaces in the retina, few cystoid spaces can be observed with the B-scan across the fovea. **B** One year after topical dorzolamide ended. Persistent cystoid spaces are observed in the en face OCT. Small cystoid spaces are also observed in the B-scan across the fovea. **C** Three months after topical dorzolamide was resumed. Regression of cystoid spaces is observed with en face OCT and B-scan

While BCVA was improved to 1.2 in the right eye and 0.7 in the left eye, en face OCT revealed residual parafoveal cystoid spaces in the left eye that persisted for 4 months after discontinuation of dorzolamide (Figs. 2A, 3A). Since these cystic spaces persisted for a prolonged duration of time and because there was a concern that they would affect vision, dorzolamide was resumed in the left eye, 1 year after the first course of topical dorzolamide treatment had ended (Figs. 2A, 3B). Three months after treatment, the cystoid spaces regressed, and the BCVA improved to 0.8 in the left eye (Figs. 2A, 3C). We ended treatment after 4 months. No recurrence was observed a year after the dorzolamide treatment ended, and BCVA improved to 1.0 in the left eye.

**Fig. 4.**
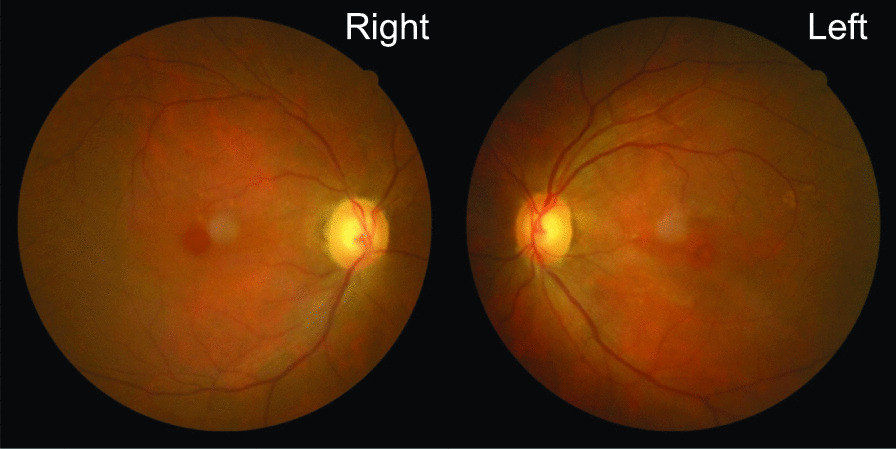
Fundus photographs in case 2, 3 weeks after the last dose of nanoparticle albumin-bound paclitaxel. No findings suggestive of vascular diseases or uveitis are observed in either fundus

**Fig. 5.**
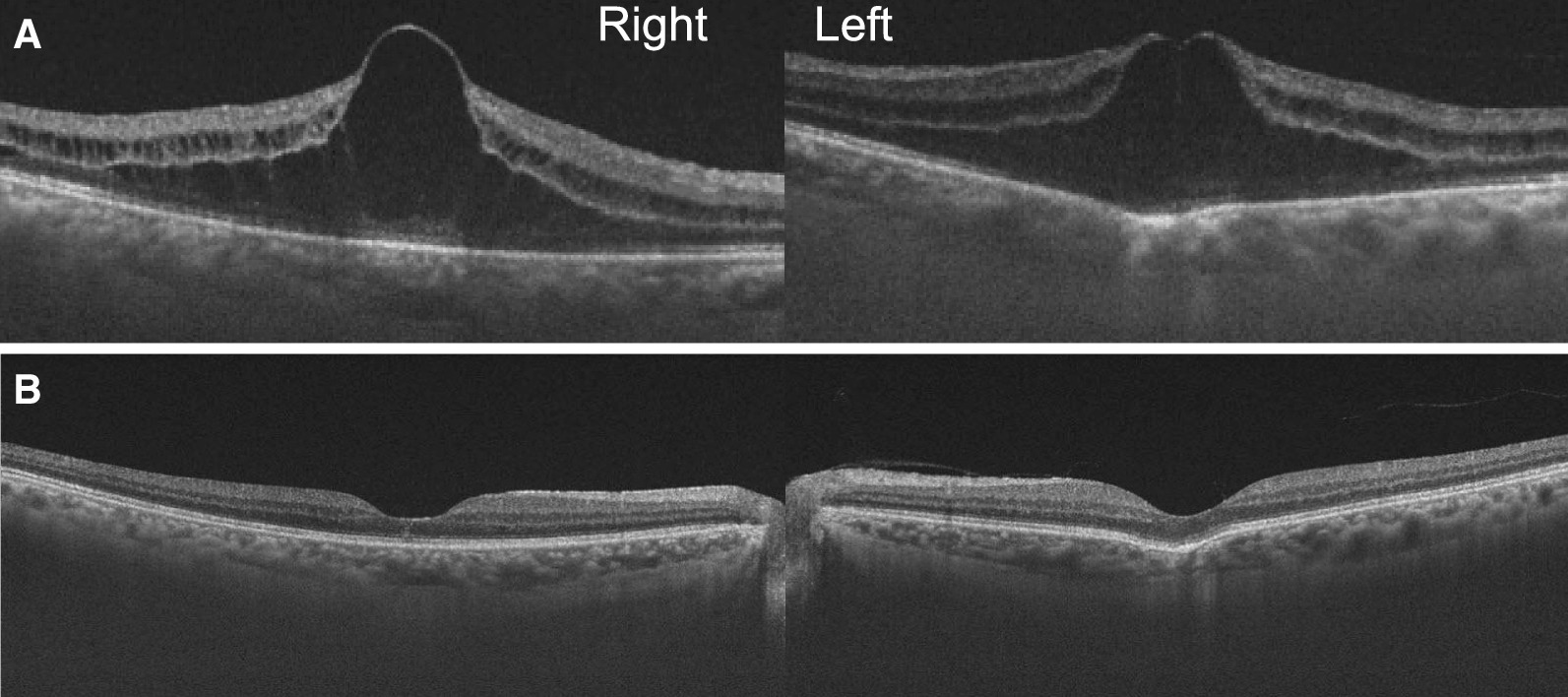
Optical coherence tomography (OCT) images in case 2 before and after topical dorzolamide treatment. **A** A 6-mm and **B** 9-mm horizontal sectional scan OCT (DRI OCT Triton, Topcon, Tokyo, Japan). **A** Three weeks after the last dose of nanoparticle albumin-bound paclitaxel treatment. OCT shows prominent cystoid macular edema (CME) in both eyes. Focal choroidal excavation is observed at the left fovea. **B** Five weeks after topical dorzolamide treatment was started. No CME is observed

Case 2: A 70-year-old Japanese man was referred to us with a 1-week history of blurred vision. He had a history of surgery for rectal cancer at 63 years of age. Five months prior, he started receiving nab-PTX plus gemcitabine therapy for pancreatic cancer with hepatic and lymph node metastases. He received 210 mg nab-PTX (125 mg/m^2^ body surface area) and gemcitabine on days 1, 8, and 15, every 4 weeks. After receiving five cycles of nab-PTX plus gemcitabine therapy, nab-PTX was terminated owing to the occurrence of sensory disorder of the limbs; only gemcitabine treatment was continued. He visited us 3 weeks after the last nab-PTX treatment. BCVA was 0.3, and the anterior chambers were clear bilaterally. Fundus examination revealed bilateral CME, and neither retinal hemorrhage nor exudate were observed (Fig. 4). Prominent CME was observed bilaterally on OCT (Fig. 5A).

We suspected taxane-related CME and initiated topical 1% dorzolamide treatment three times daily in both eyes. CME completely resolved bilaterally at his next visit at 5 weeks after starting dorzolamide treatment (Fig. 5B). Nine weeks later, his BCVA improved to 0.8 and 1.0, in the right and left eye, respectively. At this time, we terminated dorzolamide treatment. At the final visit, 2 months following discontinuation of dorzolamide treatment, there was no evidence of CME. We diagnosed this as a case of taxane-related CME, owing to the resolution of CME after cessation of nab-PTX without any treatment other than topical dorzolamide. He passed away 2 months after the final visit.

## Discussion and conclusions

In case 1, CME was successfully treated after two courses of topical dorzolamide treatment and after discontinuation of nab-PTX chemotherapy. During the first course, prominent CME resolved within 10 weeks of topical dorzolamide treatment, but a few cystoid spaces persisted for a year after cessation of the first course. These were resolved during the second course of topical dorzolamide treatment. In case 2, the CME was completely resolved within 5 weeks after starting dorzolamide treatment, and the treatment was discontinued 9 weeks later. No recurrence was observed after cessation of topical dorzolamide, and no systemic or apparent topical side effects were observed in either case.

Although initially, we could not entirely exclude the possibility of CME induced by other causes, the clinical course of CME, which regressed following initiation of topical dorzolamide and discontinuation of nab-PTX, indicated that the current cases were of taxane-related CME. Fluorescein angiography would have been performed for differential diagnosis had the CME not been resolved. Fluorescein angiography was considered unnecessary, since the patients’ histories and optical findings had provided sufficient information to start non-invasive treatments. Based on the World Health Organization-Uppsala Monitoring Centre system for standardized case causality assessment and the Naranjo algorithm (score = 6), the current cases were deemed to be “probable” taxane-related CME owing to lack of information on patient outcomes following a nab-PTX rechallenge [16, 17]. We did not consider rechallenging the patients with nab-PTX since it would not have been beneficial for the patients and would have compromised their quality of life.

The natural history of taxane-related CMEs is unknown, as only a limited number of cases have been reported. Therefore, it needs to be surveyed. There are a few published case reports on use of topical dorzolamide to treat taxane-related CME. Ehlers *et al.* (2012) reported a monocular trial of topical dorzolamide, which resulted in reduction of CME within 2 weeks, suggesting the effectiveness of the drug [11]. Hassall and Andrew (2016) reported a single-eye trial of topical dorzolamide and intravitreal bevacizumab, and the effects of both were shown to be approximately equal [15]. Dwivedi and Tiroumal (2016) reported that when topical dorzolamide treatment for taxane-related CME began 4 weeks after the patient discontinued paclitaxel, the CME resolved within 4 weeks [12]. There are a few reports on spontaneous restoration of the macular structure in taxane-related CME 3–6 months after discontinuation of the drug [6, 8, 9]. The duration of CME resolution in case 2 was 2 months from the last nab-PTX treatment, which was shorter than the reported duration of spontaneous restoration. In addition, some taxane-related CME can still persist after discontinuation of the drug, as in case 1, where a few retinal cystoid spaces persisted for a year after the cessation of the first treatment course. Topical dorzolamide appeared to accelerate CME resolution, and was also effective in treating persistent cystoid lesions induced by taxane. Systemic side effects of topical dorzolamide are rare, and common local side effects such as superficial punctate keratitis and blepharitis are treatable and are resolved with drug discontinuation [18]. Thus, we consider that a trial of topical dorzolamide should be initiated in patients with a diagnosis of clinically significant taxane-related CME.

Although evidence of the effectiveness of topical dorzolamide for taxane-related CME is still lacking, the effectiveness of topical dorzolamide in treating cystic macular spaces has been reported in X-linked retinoschisis [19, 20] and retinitis pigmentosa [21]. Both diseases show little capillary leakage on fluorescein angiography, similar to taxane-related CME. The mechanism of action of CAIs in these diseases may be the inhibition of membrane-bound carbonic anhydrase at the basolateral membrane of the RPE. This decreases the subretinal pH and enhances subretinal and intraretinal fluid resorption of the RPE, thus improving CME [20, 22].

Several hypotheses about the pathogenesis of taxane-related CME have been proposed. Taxane-related CME shows no or minimal leakage on fluorescein angiography, but molecules smaller than fluorescein may still leak and cause CME [6, 7, 14, 23]. Müller cell or RPE toxicity due to the effect of taxane on microtubules has also been proposed [14, 23–26]. Since cystoid spaces persisted, but were not worsened after discontinuation of topical dorzolamide, as suggested by en face OCT in case 1, and because cystoid spaces resolved after restarting topical dorzolamide, we speculated that taxane-related CME was not caused by subtle capillary leakage. Instead, it may be caused by an impaired retinal structure caused by taxane-induced Müller cell toxicity.

In conclusion, we demonstrated two cases in which taxane-related CME resolved after topical dorzolamide treatment and discontinuation of nab-PTX. Based on these findings, topical dorzolamide appears to be a safe and effective treatment option for taxane-related CME.

## Data Availability

The data used in the case report are available on reasonable request.

## Abbreviations

CME: Cystoid macular edema
nab-PTX: Nanoparticle albumin-bound paclitaxel
CAIs: Carbonic anhydrase inhibitors
OCT: Optical coherence tomography
BCVA: Best-corrected visual acuity
CMT: Central macular thickness
RPE: Retinal pigment epithelium
